# Secreted phospholipase A2 activity in experimental autoimmune encephalomyelitis and multiple sclerosis

**DOI:** 10.1186/1742-2094-3-26

**Published:** 2006-09-11

**Authors:** Timothy J Cunningham, Lihua Yao, Michelle Oetinger, Laura Cort, Elizabeth P Blankenhorn, Jeffrey I Greenstein

**Affiliations:** 1 Departments of Neurobiology and Anatomy, Drexel University College of Medicine, 2900 Queen Lane, Philadelphia, PA 19129, USA; 2 Department of Microbiology and Immunology, Drexel University College of Medicine, 2900 Queen Lane, Philadelphia, PA 19129, USA; 3 The Multiple Sclerosis Institute, 1740 South Street Philadelphia, PA 19146, USA

## Abstract

**Background:**

There is increased interest in the contribution of the innate immune system to multiple sclerosis (MS), including the activity of acute inflammatory mediators. The purpose of this study was to test the involvement of systemic secreted phospholipase A2 (sPLA2) enzymes in experimental autoimmune encephalomyelitis (EAE), an MS model, and to determine if enzyme activity is elevated in MS patients.

**Methods:**

A non-invasive urinary assay was developed in order to monitor enzymatically active sPLA2 levels in Dark Agouti rats after induction of EAE. Some Rats were treated with nonapeptide CHEC-9, an uncompetitive sPLA2 enzyme inhibitor, during the initial rise in urinary enzyme levels. Body weight and clinical EAE score were measured for 18 days post immunization (PI), after which the rats were sacrificed for H&E and myelin staining, and for ED-1 immunocytochemistry, the latter to quantify macrophages and activated microglia. The urinary sPLA2 assay was also applied to un-timed samples collected from a cross section of 44 MS patients and 14 healthy controls.

**Results:**

Mean levels of enzymatically active sPLA2 in the urine increased following immunization and peaked between days 8–10 PI which was just prior to the onset of EAE symptoms. At this time, a transient attenuation of activity was detected in the urine of CHEC-9 treated rats consistent with the activity-dependent properties of the inhibitor. The peptide also reduced or abolished EAE symptoms compared to vehicle-injected controls. Histopathological changes in the spinal cords of the EAE rats correlated generally with clinical score including a significant reduction in ED-1+ cells after peptide treatment. Multiple Sclerosis patients also showed elevations in sPLA2 enzyme activity. Mean levels of sPLA2 were increased 6-fold in the urine of patients with active disease and 4-fold for patients in remission, regardless of immunomodulating therapy.

**Conclusion:**

The results suggest that sPLA2 enzymes, traditionally thought to be part the acute phase inflammatory response, are therapeutic targets for MS.

## Background

The pathophysiology of multiple sclerosis (MS) involves both antigen specific mechanisms and the innate immune system, including elements of the acute inflammatory response [1-4]. Since axonal loss is likely to begin at disease onset, the inflammation that accompanies this degeneration may be a persistent contributing factor in MS, as it all disorders in which there is destruction of nervous tissue. Increased hydrolysis of membrane phospholipids by phospholipase A2 is a well-known early response to tissue damage in all organ systems including the nervous system [5-7]. These enzymes are responsible for the production of arachidonic acid and lysophospholipids and therefore also control levels of inflammatory mediators and cytotoxic metabolites including prostaglandins, leukotrienes, and reactive oxygen species. PLA2 enzymes and products also influence several aspects of the cellular and cytokine involvement in the inflammatory response.

Recent studies of rodent experimental autoimmune encephalomyelitis (EAE) models of MS suggest PLA2 enzymes are involved in the onset and genesis of this disease [8,9]. Pinto, et al.[8], found that systemic infusion of anchored lipid conjugates, targeting extracellular or secreted (s)PLA2s, attenuated aspects of the autoimmune response and EAE clinical disease. We have identified a nonapeptide sPLA2 inhibitor called CHEC-9 that has properties that may be particularly advantageous for applications *in vivo *[32]. CHEC-9 is an internal fragment of the endogenous protein DSEP/Dermcidin/PIF [10-16]. Both the parent polypeptide and isolated peptide mimetics, including CHEC-9, have been used previously to increase cell survival and inhibit inflammation in a variety of experimental paradigms.

In the present study, we used a non-invasive urinary assay to monitor changes in systemic levels of active sPLA2 enzymes following a standard EAE immunization protocol. We also examined the effects of CHEC-9 on the development of EAE symptoms, including correlated morphological changes in the spinal cord. Finally, this same urinary assay was applied to randomly collected urine samples from MS patients to determine if systemic levels of enzyme are also elevated in this disease.

## Methods

### EAE production and analysis

The animal experiments were conducted under the auspices of an IACUC protocol from Drexel University. All personnel involved were experimentally blinded for all procedures. Mild to moderate EAE was induced in 20 female Dark Agouti rats (130–150 g) by bilateral foot pad immunizations of 100 μg guinea pig myelin basic protein in saline emulsified with Complete Freund's Adjuvant [17]. The results presented are from 2 separate sets of experiments with 10 in each cohort, equally divided between peptide and vehicle-treated rats. The rats were weighed and scored for EAE symptoms daily using a 1–4 rating scale for clinical disease (1-tail drop, 2 hind limb paresis, 3 hind limb paralysis, 4 moribund), and 2–3 independent investigators were responsible for the scoring. We scored 2.5 for complete paralysis of one hind limb which was the most severe disease encountered in this experimental system. The experiments lasted 18 days following immunization, after which the rats were perfused with 4% paraformaldehyde in 0.1 M phosphate buffer and their spinal cords removed for histology.

### Urine collection and CHEC-9 treatment

Urine was collected in metabolic cages from all rats starting 1–2 days before immunization and then every other day for 18 days. The urine was collected between 9:00–11:00 AM, immediately sterile filtered, and frozen at -40° prior to use. CHEC-9 treatment started 5 days after immunization during the rise in urinary sPLA2 activity (see Results). Treatment consisted of a subcutaneous injection of 60μg CHEC-9 in clear DMEM vehicle on the first day, followed by daily 30μg doses for 9 days. CHEC-9, CHEASAAQC, was made by Celtek (Nashville, TN), purified and cross-linked as described previously[10].

### sPLA2 enzyme activity

Twenty-five μl samples of urine were reacted with 600μM 1,2-*bis *(heptanoylthio) glycerophosphocholine, a substrate for all PLA2s with the exception of cPLA2 and PAF-AH (Caymen Chemical, Ann Arbor MI). Reaction buffer consisted of 50 mM tris, 0.1 M NaCl (pH = 7.4) containing 1 mM DTNB (Ellman's reagent) and 2 mM CaCl_2_. Reaction rates were determined with a Deltasoft (Princeton NJ) supported ELX 808 reader (Biotek, Burlington VT). The urinary sPLA2 assay was developed because in a previous study we found that monitoring active enzyme in blood plasma may be subject to considerable variability due to the stress of restraint and collection procedures, as well as handling of samples for *ex vivo *monitoring [32]. Enzyme activity in urine was found to be much more stable, specifically in those rats tested prior to immunization or in healthy human controls. As in previous studies in which the active enzyme concentration was of interest, we confirmed that product formation in the presence of an excess of substrate was linear during a 40 minute measurement period, and therefore that the measured rate of the reaction is proportional to urinary concentration of active sPLA2. These rates were expressed relative to total protein in the samples and normalized either to average baseline values for the rats (obtained in the two days prior to immunization), or in the patient studies, to average value obtained from healthy controls.

### Statistical tests

Data comparisons were by a two-tailed Mann Whitney test, nonparametric (Spearmann) linear correlation, or Friedman's repeated measures ANOVA.

### Histology and immunohistochemistry

Serial 20μm transverse (n = 10) or parasagittal sections (n = 10) were cut through spinal cord blocks that extended from the conus medullaris to the lumbar enlargement. Alternate transverse sections were stained with hemotoxylin & eosin and cyanin R the latter for myelin and myelin debris (e.g. see ref. [18]). The parasaggital sections allowed for greater areal coverage of this part of the spinal cord and therefore these were used for quantification of macrophages, ameboid microglia, and activated microglia using the ED-1 monoclonal antibody (Serotec, UK). The cells were counted by experimentally blinded investigators in 5-vehicle treated rats and 5 peptide-treated rats using three equivalent parasaggital sections from each rat. The counts were expressed per area of the sections and then normalized to the average value obtained in the vehicle-treated rats.

### Patients

Forty-four patients with a diagnosis of relapsing/remitting Multiple Sclerosis provided urine samples for this study (Table 1). Healthy controls were recruited by advertisement. The Institutional Review Boards of Drexel University and Graduate Hospital of Philadelphia approved the study and informed consent was obtained from all subjects. Twelve of the patients presented with active disease at the time of sample collection, which was defined as a change of one or more points on the functional neurologic status score in the absence of fever or infection [19]. Therapies noted in Table 1 were interferon beta-1a (Avonex^®^, Biogen, 30μg/week I.M.) or glatiramer acetate (Copaxone^®^, Teva Pharmaceutical Industries 20 mg/day, S.C.). Subjects were carefully screened and those with peripheral infections, inflammatory disorders, or those using anti-inflammatory drugs within the 24 hours prior to sample collection, were excluded from the study. Urine was collected in a sterile 50 ml tube at random times during the day (9:00 AM-4:00 PM), immediately sterile filtered, frozen and analyzed as above.

**Table 1:** Patient population and control subjects used for the sPLA2 measurements.

**Group**	**n (F/M)**	**Age yrs ± sd**	**MS Onset yrs ± sd**	**MS Duration yrs ± sd**	**βIF**	**GA**	**None**
Active	12 (8/4)	38.2 ± 9.5	29.5 ± 8.9	7.3 ± 4.5	9	1	2
Stable	32 (25/7)	43.6 ± 8.1	33.4 ± 8.6	10.6 ± 9.3	15	3	14
Control	14 (9/5)	37.5 ± 9.0	-	-	-	-	-

The means of subject age, age of MS onset, duration of the disease, and ongoing therapies are listed. F/M = female/male; βIF = beta-interferon; GA = glatiramer acetate; sd = standard deviation.

## Results

### Body weight

There was an initial loss of body weight in all rats post-immunization (PI). From PI day 5 (the start of treatment) onward, peptide treated animals gained weight at a significantly higher rate than vehicle treated rats. Weight gain in the peptide-treated animals was 0.517 g/day compared to 0.392 g/day in vehicle-treated rats. The difference between groups was significant (p = 0.011, Friedman's repeated measures ANOVA).

### sPLA2 activity and clinical score

**Figure 1:**
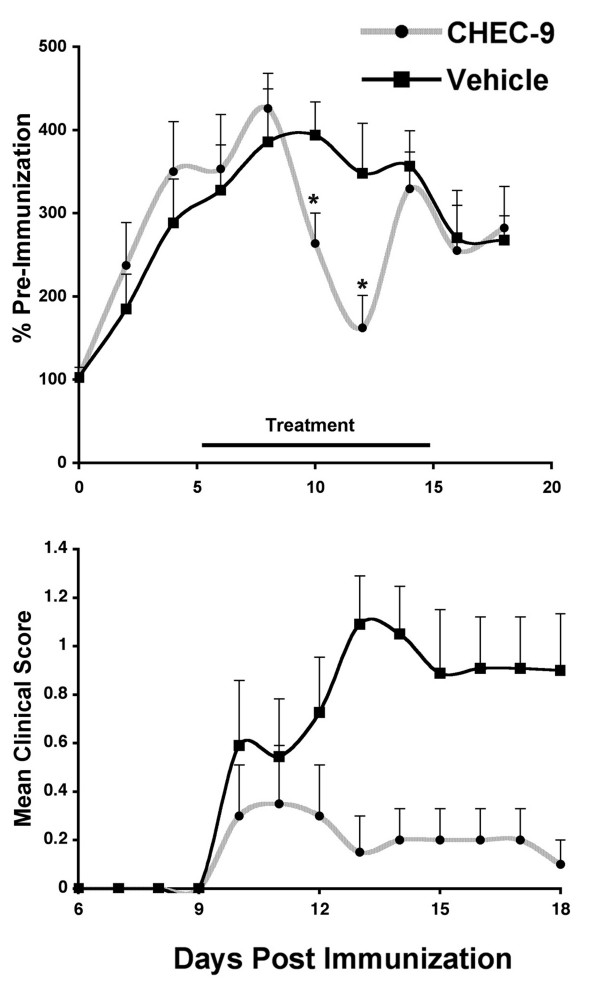
**Secreted phospholipase A2 (sPLA2) activity and clinical disease in EAE rats treated with sPLA2 inhibitor CHEC-9**. **Top**: Urinary sPLA2 enzymatic activity, normalized to average pre-immunization values, increased steadily to day 8 in both CHEC-9 and vehicle-treated rats. A significant reduction in activity was observed on days 10 and 12 post-immunization in the peptide treated group either by comparing values of peptide and vehicle directly (p = 0.049, 0.026 respectively, Mann Whitney), or by peak to trough comparison between days 8 and 12, where reduction in sPLA2 levels with peptide treatment was significant (p = 10^-3^). **Bottom**: Mean clinical scores from day 10 onwards were also significantly lower in the peptide treated rats (see text).

Mean urinary sPLA2 activity increased steadily for the first 8 days following immunization in both treatment groups (Fig. 1, Top). In vehicle treated rats, the mean activity leveled off and declined at this point. The effects of CHEC-9 treatment were not detected until days 10 and 12 PI, when the peptide significantly attenuated sPLA2 activity. It was during this period that the behavioral deficits appeared in the rats (Fig. 1, Bottom). However, only 3/10 peptide-treated rats showed symptoms of disease and one of these had a late onset tail paresis (at day 17 post-immunization). This compared to 8/10 in the vehicle treated group showing generally more severe symptoms that appeared between days 10 and 13 PI. As might be expected from these differences, the statistical difference between the two groups was very significant (p = 0.0002, Friedman's repeated measures ANOVA).

### Histology and immunohistochemistry

**Figure 2:**
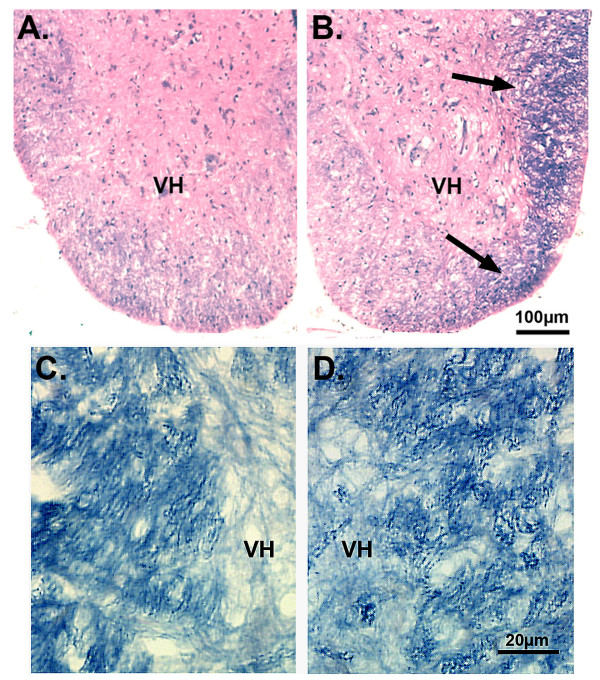
Low power photomicrograph of hematoxylin & eosin (A, B) and cyanin R (C, D) stained sections through the lumbar spinal cord. Left panels (A, C): Spinal cord of a peptide-treated rat that was symptom-free showing limited small cell infusion and myelin degeneration. Right panels (B, D): Spinal cord of a vehicle treated rat that had an EAE score of 2.0. The tissue was characterized by EAE lesions consisting of small darkly staining cells in the ventral and lateral white matter (arrows), and myelin degeneration as shown in an adjacent section (D).

**Figure 3:**
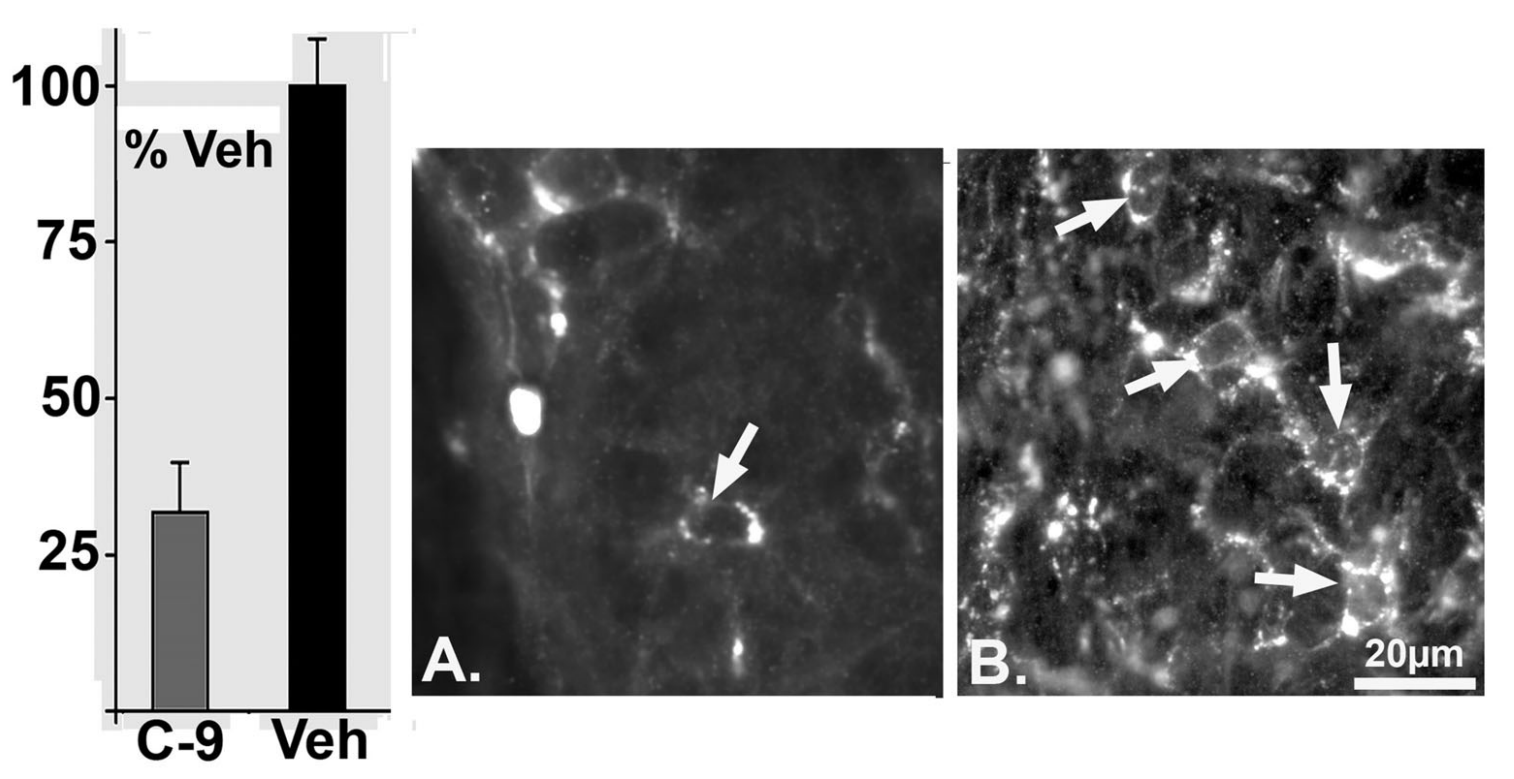
Macrophages and microglia were reduced by CHEC-9 treatment of EAE. Graph (left panel): The density of ED-1+ cells in the spinal cord was reduced over 60% following peptide treatment (p < 1 × 10^-3^). Although the immunostained cells tended to accumulate at or near the surface of both groups, fewer cells, occupying a smaller area within the cord were found CHEC-9 treated rats (B) compared to vehicle treated rats (C). The majority of intraspinal ED-1 immunoreactive cells were activated microglia (arrows).

Hematoxylin & eosin together with cyanin R staining in alternate sections of the spinal cord revealed pathology consistent with the clinical score. The most conspicuous changes appeared in rats showing the most severe deficits (clinical score 2.0 or greater). In the caudal-most regions of the cord of these rats, the region around the conus medullaris was severely shrunken and contained large areas of degenerating myelin. In more rostral sections, large EAE lesions were found often localized to the white matter (Fig. 2). Adjacent sections stained for cyanin R revealed that these areas were also characterized by dense myelin debris (Fig. 2). Symptom-free rats had limited and more scattered small cell infusion and myelin debris. To quantify the immune response, we determined the number of macrophages and microglia by counts of ED-1+ cells within the spinal cord. The counts were made in representative vehicle-treated rats (n = 5, mean clinical score, 1.6) and peptide-treated rats (n = 5, mean clinical score, 0.2). Both groups of rats showed immunoreactive cells on the spinal cord surface with a variable numbers of cells occupying sub-adjacent white matter and parenchyma. The ED-1+ cells included large round macrophages, smaller round ameboid microglia, and "activated" microglia, which were large (presumably hypertrophied) cells with processes. The latter cell type dominated the tissue especially in the vehicle treated rats (Fig. 3). In fact, CHEC-9 treated rats had over 60% fewer ED-1+ cells per unit area of the sections (p < 1 × 10^-3^, Mann Whitney test). In addition, there was an obvious difference in the extent to which the cells had infiltrated the spinal cord in the control-treated rats. However, the calculated difference in density may be inflated because in two of the vehicle treated rats used for this part of the study, there was shrinkage of the caudal spinal cord (EAE scores 2.0, 2.5). Nevertheless, these results showed clearly that clinical EAE score reflected the histopathological changes in the spinal cord, as has been described in numerous other studies with the EAE model [20]. The results also suggested that the peptide's therapeutic effects included inhibition of some aspects of the cellular immune response, as has been shown previously after traumatic CNS lesions [10].

### MS patients

**Figure 4:**
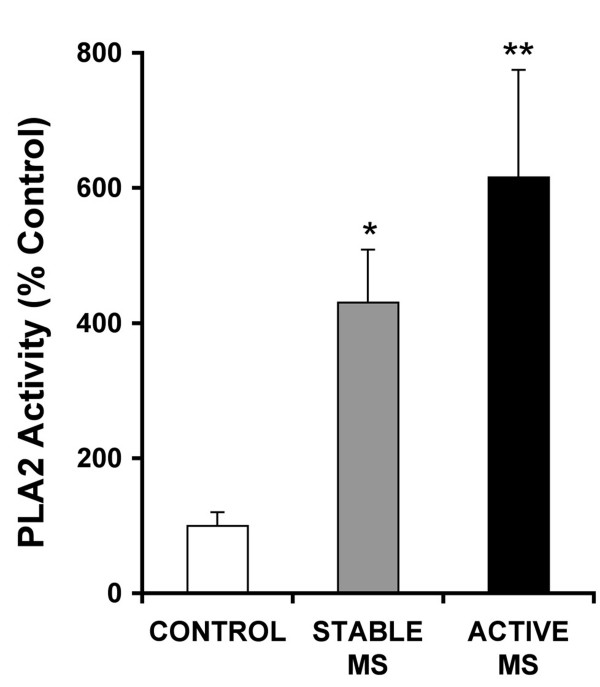
**Level of sPLA2 enzyme activity in MS patients with active or stable disease compared to controls (see Table 1)**. All measurements were made using 600 μM substrate and normalized to the average control value. There was a significant 4 and 6-fold increase in PLA2 activity compared to controls in the stable and active MS patients respectively, (p = 0.049*; 0.0019**, for comparison with controls, Mann Whitney test). Treated and untreated stable patients were grouped since their average levels of active enzyme were almost identical (see text). Patients with active disease were mostly undergoing treatment with beta-interferon (9/12, Table 1).

The mean level of active sPLA2 enzymes was significantly elevated in the urine of patients with both active and stable MS (Fig. 4). Enzyme concentrations were higher on average in relapsing patients compared to non-relapsing patients although the difference did not reach statistical significance (p = 0.107, Mann Whitney test). Interestingly, the increased concentration of enzyme found in stable patients receiving no treatment was nearly identical to that from patients treated with beta interferon (mean 393 ± 130% of controls n = 15, versus 409 ± 98% s.e.m., n = 14, p = 0.585, Mann Whitney test), although both these patient populations were somewhat heterogeneous as shown by the large variances.

In order to rule out differences in renal function between the groups, we compared total urinary protein with sPLA2 enzyme levels. Enzyme and protein levels were not correlated in any group (p values for protein-sPLA2 correlation, Active = 0.572; Stable = 0.350; Control = 0.885, Spearmann's nonparametric linear correlation). Therefore, it was unlikely that the patients simply leaked more sPLA2 because of a systematic and unrecognized renal/urinary tract pathology associated with MS. Notably, untimed 'spot' samples as used in this study are routinely applied and accepted as an indicator of excess protein in the urine (proteinuria) [21], which is expected to correlate with elevated PLA2 enzyme levels under conditions of compromised renal function [22,23].

## Discussion

The present results suggest that increased levels of sPLA2 enzymes, long associated with inflammation outside the nervous system[24], also characterize Multiple Sclerosis and Experimental Autoimmune Encephalomyelitis. Although the EAE model has been questioned recently in terms of its validity for identifying potential MS therapies [25,26], the model is clearly useful to help define the role of inflammatory enzymes, specifically the PLA2s, in autoimmune attack of the nervous system. Using a simple urinary assay for active enzyme concentration, we found that rats immunized to produce EAE had evidence of increased systemic sPLA2 following immunization. The same assay applied to samples collected from MS patients also showed increased levels of active enzyme even with random or "spot" sampling. Based in part on the parallel results in the rodent model, we conclude that sPLA2 inflammatory activity is ongoing in the majority of MS patients, active or stable, regardless of treatment. The highest levels of enzyme were found in patients with active disease, i.e., during relapse. Interestingly, asymptomatic EAE rats had elevated enzyme activity but became symptomatic only after a peak in activity was detected. Finally, and most important, EAE symptoms were attenuated by sPLA2 inhibitor CHEC-9. This finding, along with similar results by others [8,9], support the idea that PLA2 enzymes play a direct role in the pathogenesis of MS and related autoimmune disorders.

It important to recognize that increased PLA2 activity is found after a variety of inflammatory stimuli, from localized tissue damage to systemic infections [32]. In addition, a variety of antigenic stimuli are expected to raise systemic sPLA2 levels, which suggests the nature of the antigen is also critical in determining the resulting pathology. On the other hand, the contribution of some types of peripheral or systemic infections to induction and exacerbation of autoimmune disorders, including those affecting the nervous system, is well known [27,28]. This non-specific regulation of specific autoimmune disorders, or cross talk between innate and acquired immune responses, could depend in part on acute phase mediators like sPLA2. A key element in this interaction may be lysophosphatidyl choline, an important but often overlooked product of sPLA2 activity. LysoPC has been implicated in several aspects of acquired immunity including lymphocyte and monocyte chemotaxis, as well as dendritic cell differentiation [29-31].

The appearance of symptoms at or shortly after the peak of sPLA2 activity is interesting, especially if this peak reflects maximal levels of inflammation and oxidative stress. For example, it is under these conditions that sPLA2 may exert the most profound effects on the activity of cytosolic PLA2 (cPLA2 [32]). The specific involvement of cPLA2 in EAE has also been demonstrated [9]. It is also under such conditions that the sPLA2 inhibition by the peptide is expected to be maximal because of its properties as an uncompetitive inhibitor, including activity-dependent inhibition [32]. These properties may also explain our ability to detect a significant attenuation of enzyme activity in urine of peptide treated rats only at this time but not before or after, even with peptide treatment. However, measurements every 48 hours are not likely to be sensitive enough to reveal the actual pharmakinetics of CHEC-9, which is currently under study. Therefore the peptide's full effects on enzyme activity during the course of these experiments are unknown. In addition, small intermittent reductions of activity, that may go undetected in urine, could be sufficient to slow or even prevent the onset or appearance of more severe EAE symptoms.

The above discussion of the therapeutic effects of CHEC-9 assumes that the peptide's effects are due to enzyme inhibition, i.e., decreases in sPLA2-mediated inflammation and in the inflammatory products of PLA2-driven metabolism, directly and via cross talk with cPLA2. This explanation is especially attractive since CHEC-9 is the third of three different PLA2 inhibitors that has been used to treat the disease. However, there are other properties of these enzymes, including their direct influence on neuron viability. For example, sPLA2 enzymes potentiate excitoxicity, a mechanism suggested to be involved in most instances of neurodegeneration [33, 34]. Also, as noted above, there are likely to be interactions of the PLA2 enzymes with elements of antigen-specific immunity. Furthermore, sPLA2s influence levels of proinflammatory cytokines in a variety of cell types (either directly or via cPLA2), especially during periods of oxidative stress [24, 32, 35, 36, 37]. In some instances this influence does not depend on enzymatic activity. Thus, sPLA2 enzymes may be an important therapeutic target for a variety neurodegenerative disorders associated with exaggerated autoimmune or inflammatory reactions, and especially for diseases such as MS, where elements of both processes contribute to pathology.

## Conclusion

Further study of sPLA2-regulated processes in EAE models may provide new insights into therapies for autoimmune disorders affecting the nervous system. Amelioration of EAE by uncompetitive sPLA2 inhibitor CHEC-9 suggests a direct role for these enzymes in such disorders. Furthermore, monitoring sPLA2 activity in MS patients, for example in relation to their susceptibility to relapse, could help define periods of vulnerability in these patients as well as appropriate regimens for application of therapies involving sPLA2 inhibition.

## Competing interests

TJC, LY, and Drexel University have applied for patent protection of CHEC-9, a peptide inhibitor used in these experiments.

## Authors' contributions

TJC drafted the manuscript, organized the assay data for computer analysis and participated in the histological analysis. LY carried out the enzyme assays, immunohistochemistry, and assisted in the EAE studies. MO assisted in the histological analysis, EAE studies, and in the patient studies. EB criticized the manuscript and together with LC designed and implemented the EAE studies. JG screened subjects for the human studies, conducted neurological exams and collected samples from patients and controls. He also assisted in data interpretation, and in preparing the manuscript. All authors approved of the final version of the manuscript.
